# Innovative Techniques to Enhance Musculoskeletal Surgery Outcomes

**DOI:** 10.1155/2018/7189240

**Published:** 2018-11-13

**Authors:** Berardo Di Matteo, Peter Angele, Christian Lattermann, Elizaveta Kon

**Affiliations:** ^1^Humanitas University Department of Biomedical Sciences, Via Manzoni 113, 20089 Rozzano, Milan, Italy; ^2^Humanitas Clinical and Research Center, Via Manzoni 56, 20089 Rozzano, Milan, Italy; ^3^Department of Trauma Surgery, University Medical Center Regensburg & Sporthopaedicum Straubing, Regensburg, Germany; ^4^Brigham and Women's Hospital, Harvard Medical School, Chestnut Hill, MA, USA

In the last 15 years, orthopaedic practice has been revolutionized by the amazing progresses made in the field of biotechnologies, which led to the introduction of innovative therapeutical approaches aimed at minimizing surgical distress for patients and, at the same time, providing better healing chances for many musculoskeletal diseases [1]. Beside the great enthusiasm for novel strategies represented by the use of biomaterials and biologic agents, such as autologous growth factors [2] and stem cells [3], there has been also an increasing awareness on the necessity of better understanding the mechanisms of pathology, improving our diagnostic skills, and identifying positive and negative prognostic factors [4]. In fact, not only is the “therapeutic success” based on the treatment itself, but it is the result of a more complex interaction of factors: an early diagnosis, a proper timing for starting the treatment, and the specific patient features which could determine either a satisfactory or poor outcome. This multifactorial approach is usually regarded as “patient profiling” and represents the pinnacle of the “personalized medicine” which is the ultimate goal of current research in all medical specialties [5]: providing a treatment “tailored” to the specific needs of the patients in order to maximize the benefit and reduce potential side effects. Therefore, the focus of clinicians has shifted from the pathology as a stand-alone entity to the concept of “pathology within a specific patient”: based on this conception, it is not only the disease that changes the “homeostasis” of the patient, but the patient himself can influence the course of the pathology in a favourable or negative manner, depending on his/her particular features [6, 7]. The challenge is always to understand how to take advantage of the “pros” and how to get rid of the “cons.”

This was the idea that led us to propose the present special issue, whose title reflects the curiosity of all the guest editors towards the potential ways to enhance the outcomes of musculoskeletal surgery. The title is very broad, and we expected contributions coming from very different areas, ranging from basic traumatology to elective surgery and also preclinical experiments. This was a conscious choice, since we believe that sharing ideas from a wide range of different clinical scenarios could be a winning method to provide fruitful stimuli to the readers, avoiding excessive subspecialization: sometimes to find solutions you have to raise your eyes from the microscope and take a wider look around you. We believe that innovative strategies in one particular field might perhaps be applied in completely different situations, and therefore it would have been interesting to include in the same special issue perspectives coming from basic researchers, traumatologists, spine surgeons, sports medicine surgeons, and so on. We are truly glad to have received such a great interest, which is testified by a total of 15 papers accepted. Looking at the topics proposed in the whole issue, four main areas can be identified: (1) biomarkers and molecular pathways (for early diagnosis or as potential therapeutic target); (2) computer aided surgery and patient-specific implants; (3) biomaterials and biologic agents for tissue regeneration; (4) evaluation of prognostic factors after surgery. All the major fields related to the “personalization” of the treatment have been covered, with very interesting contributions. Regarding biomarkers and molecular pathways, four papers have been included in the issue: one investigated the role of serum biomarkers in predicting heart-related complications following hip fracture, one dealt with synovial procalcitonin for the detection of periprosthetic joint infection, one focused on ACL reconstructive surgery, and, lastly, there was an interesting review on the role of Wnt-pathway in the pathogenesis of OA. Looking at computer aided surgery and patients' specific implants, we have papers dealing with innovative frames for pelvic fracture stabilization and 3D-printed PEEK hardware. In the field of biomaterials and regenerative medicine we included an animal trial on Achilles tendon collagen scaffold and a clinical trial investigating the potential of PRP in stimulating healing of partial ACL rupture. Considering papers on “prognostic factors,” we included one multicentric trial presenting a score to predict tibial fracture healing time, one systematic review on radial head fractures, one retrospective trial investigating the failure predictors in pediatric forearm fractures, and one pilot study which revealed particular histological features of the articular capsule of patients affected by glenohumeral instability.

Perhaps some readers will be bewildered by the variability of the topics included, but we believe this is actually the strength of our issue, which offered a panoramic overview on the many different innovative approaches to the treatment of disparate musculoskeletal conditions. Therefore, it should be considered a starting point for future, more focused insights on specific pathologies, in the attempt of strengthening the conviction that the outcomes of innovative treatments are strictly related to the timing of diagnosis and the patients' intrinsic features. The mere “technological” improvement will not provide better results if not accompanied by the understanding of the disease mechanisms and the factors playing a crucial role in its progress.

## References

[1] Di Matteo B., Perdisa F., Gostynska N., Kon E., Filardo G., Marcacci M. (2015). Meniscal Scaffolds - Preclinical Evidence to Support their Use: A Systematic Review. *The Open Orthopaedics Journal*.

[2] Filardo G., Kon E., Di Matteo B. (2014). Leukocyte-poor PRP application for the treatment of knee osteoarthritis. *Joints*.

[3] Zellner J., Pattappa G., Koch M. (2017). Autologous mesenchymal stem cells or meniscal cells: what is the best cell source for regenerative meniscus treatment in an early osteoarthritis situation?. *Stem Cell Research & Therapy*.

[4] King J. D., Rowland G., Villasante Tezanos A. G. (2018). Joint Fluid Proteome after Anterior Cruciate Ligament Rupture Reflects an Acute Posttraumatic Inflammatory and Chondrodegenerative State. *Cartilage*.

[5] Alyass A., Turcotte M., Meyre D. (2015). From big data analysis to personalized medicine for all: challenges and opportunities. *BMC Medical Genomics*.

[6] Selmi C., Kon E., Santis M. D. (2018). How advances in personalized medicine will change rheumatology. *Personalized Medicine*.

[7] Borro M., Simmaco M., Aceti A. (2016). H2020 and beyond: Skip discrepancy between theory and practice of personalized medicine. a position paper by the Italian society of personalized medicine. *Current Pharmaceutical Biotechnology*.

